# Management of acute COPD exacerbations in France: A qualitative survey in a private practice setting

**DOI:** 10.1371/journal.pone.0245373

**Published:** 2021-01-22

**Authors:** Anne Compagnon, Laurent Nguyen, Zeina Antoun, Bruno Stach, Christophe Zanetti, Frédéric Le Guillou

**Affiliations:** 1 Medical Department, GlaxoSmithKline, Rueil-Malmaison, France; 2 Association de réflexion sur le Parcours de Soins en milieu Respiratoire, La Rochelle, France; 3 Global Specialty and Primary Care, GlaxoSmithKline, Uxbridge, United Kingdom; BronxCare Health System, Affiliated with Icahn School of Medicine at Mount Sinai, UNITED STATES

## Abstract

**Background:**

The current prevalence of chronic obstructive pulmonary disease (COPD) in France is estimated to be 2.6 million and is predicted to increase to 2.8 million by 2025. Presently, there is a lack of data on COPD management within the private healthcare setting. The aim of this study was to investigate the management of COPD exacerbations by pulmonologists within private practices in France.

**Methods:**

A prospective, online, qualitative survey was distributed to private practice pulmonologists in France. The survey covered all aspects of COPD management from diagnosis and therapeutic management, to secondary prevention and organization of care. Survey responses were collected between 27 January 2018 and 18 June 2018 and all data were summarized descriptively.

**Results:**

The survey had a response rate of 20.6%, with 116 out of 563 pulmonologists providing responses. Overall, 87.4% of respondents stated that the management of COPD represented over 15% of their total clinical activity. Most respondents indicated that they work closely with general practitioners and a large multidisciplinary team to manage patients with numerous comorbidities. Following a COPD exacerbation, the majority of respondents (78.4%) were in favor of using respiratory-connected devices (class 2a-connected medical device according to the French HAS classification and available on medical prescription) to assist with patient follow-up at home.

**Conclusions:**

COPD management forms part of the core clinical activity for pulmonologists within the private practice setting in France. Patients with COPD generally have multiple comorbidities and are managed by a multidisciplinary team in line with French guidelines. The use of respiratory-connected devices was highlighted as an important new strategy for improving patient care following a COPD exacerbation.

## Introduction

Chronic obstructive pulmonary disease (COPD) is a common, preventable, and treatable disease characterized by airflow limitation and persistent respiratory symptoms [1]. The global burden of disease for COPD is substantial, with the World Health Organization reporting it to be in the top 5 leading causes of death worldwide in 2016 [2], and projections indicating that it will remain a leading cause of death by 2040 [3]. The prevalence of COPD in France is currently estimated to be over 2.6 million and is predicted to increase to over 2.8 million by 2025 [4].

The goals of care for COPD management include reducing symptoms, improving health status and exercise tolerance, and reducing the risk of COPD exacerbations (periods of acute worsening of symptoms) to ultimately prevent disease progression and reduce mortality [1]. Acute exacerbations negatively impact health status, accelerate lung function decline, and increase mortality risk [1, 5]. Overall, they account for more than half the hospitalizations in COPD [5]. In France, a retrospective study of administrative records between 2007 and 2012 revealed that of 29,779 patients admitted to hospital with a severe exacerbation, over 8% died during hospitalization [6]. A separate, prospective study of 1,750 patients admitted to French general hospitals for acute COPD exacerbations between October 2006 and June 2007 reported that 787 (45.0%) died within 48 months of admission [7]. As such, COPD exacerbations represent a key therapeutic target in COPD and recognizing the impact of COPD and putting a management strategy in place is an important topic in France [8]. Currently, the Global initiative for chronic Obstructive Lung Disease (GOLD), Haute Autorité de Santé (HAS) and Société de Pneumologie de Langue Française (SPLF) recommend similar approaches for the management of COPD exacerbations. These include bronchodilation (short-acting inhaled β_2_-agonists with or without short-acting anticholinergics), oral corticosteroids, oxygen supplementation, antibiotics (when signs of bacterial infection are present), and non-invasive mechanical ventilation (in patients with acute respiratory failure and no contraindications) [1, 9].

Strategies for the development of personalized care plans for patients with COPD are continually evolving. These include the promotion of outpatient care, the reassessment of patients within a 24–72-hour window to avert hospital readmissions and the development of home-based care for patients with COPD [10, 11]. In particular, the Programme d’Accompagnement de Retour à Domicile (PRADO-BPCO) was set up in 2015 by the French National Health system [12]. This program comprises a home discharge care package with the aim of reducing readmission rates of patients with COPD following an acute exacerbation [12].

While studies have previously investigated the management of exacerbations as part of clinical trials and in the public healthcare setting, there is currently a lack of data within the private healthcare setting. In France, most studies on the management of acute COPD exacerbations are carried out within public hospital-based settings with established patient care services within the same hospital. However, unlike many countries, specialist care for patients with COPD in France is not exclusively hospital based. French patient care pathways often integrate private patient care in an outpatient care setting connecting a number of healthcare professionals, such as general practitioners and pulmonologists operating at local levels. According to figures from DREES in 2018, 1274 out of 3090 pulmonologists operated in this manner [13]. To date, few studies have described the private healthcare pathways for patients with COPD in France and their links to more established hospital-based care services.

The aim of this study was to investigate the management of COPD in the private practice setting in France using a prospective, online, qualitative survey aimed at practicing pulmonologists, which covered all aspects of COPD management from diagnosis and therapeutic management, through to secondary prevention and organization of care.

## Methods

The online survey was developed using the SurveyMonkey^®^ platform (SurveyMonkey, San Mateo, CA, USA) and distributed to all 563 pulmonologist members of the Association de reflexion sur le Parcours de Soins en milieu Respiratoire (APSR) via email between 27 January 2018 and 18 June 2018. All members were private practice pulmonologists located in France. According to recent DREES figures there were 1274 pulmonologists operating in private practices in France [13]. As such, the 563 APSR members who were surveyed were considered to be representative of pulmonologists working in the private practice setting in France.

The questionnaire consisted of four main sections: respondent profile, place of COPD management in clinical practice, patient care pathway for COPD exacerbations and secondary prevention. Specific information obtained via the questionnaire included: the age of respondent, type of practice and location, number of patients typically seen each month with COPD/COPD exacerbations, proportion of time spent managing patients with COPD/COPD exacerbations, treatment and referral locations, etiological assessments, first-line therapies used and measures of secondary prevention and follow-up.

The definition of a respiratory-connected device was any class 2a-connected medical device according to the French HAS classification and available on medical prescription. Examples of these devices include smartwatches and smart bracelets capable of remotely monitoring key vital signs within patients such as oxygen saturation, heartbeat and electrocardiogram.

Survey responses were tallied and totaled. Percentages were then calculated based on the total responses received. All data were summarized descriptively.

### Ethics approval and informed consent

Ethics approval and patient consent were not required, as this was a qualitative survey of procedures employed by pulmonologists in private clinical practice.

## Results

### Respondent profile

**Fig 1:**
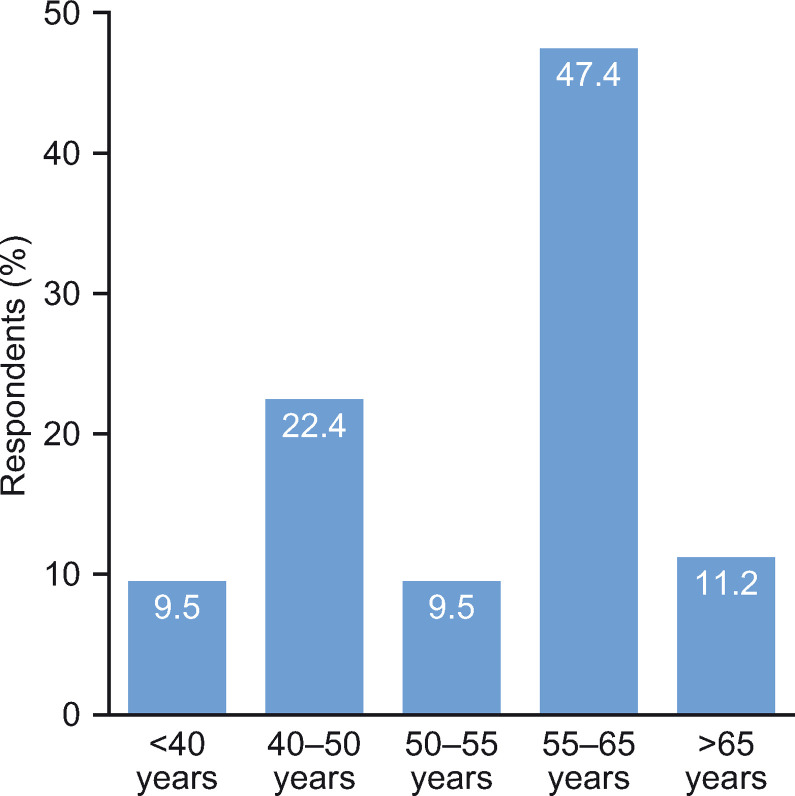
Age range of respondents.

**Fig 2:**
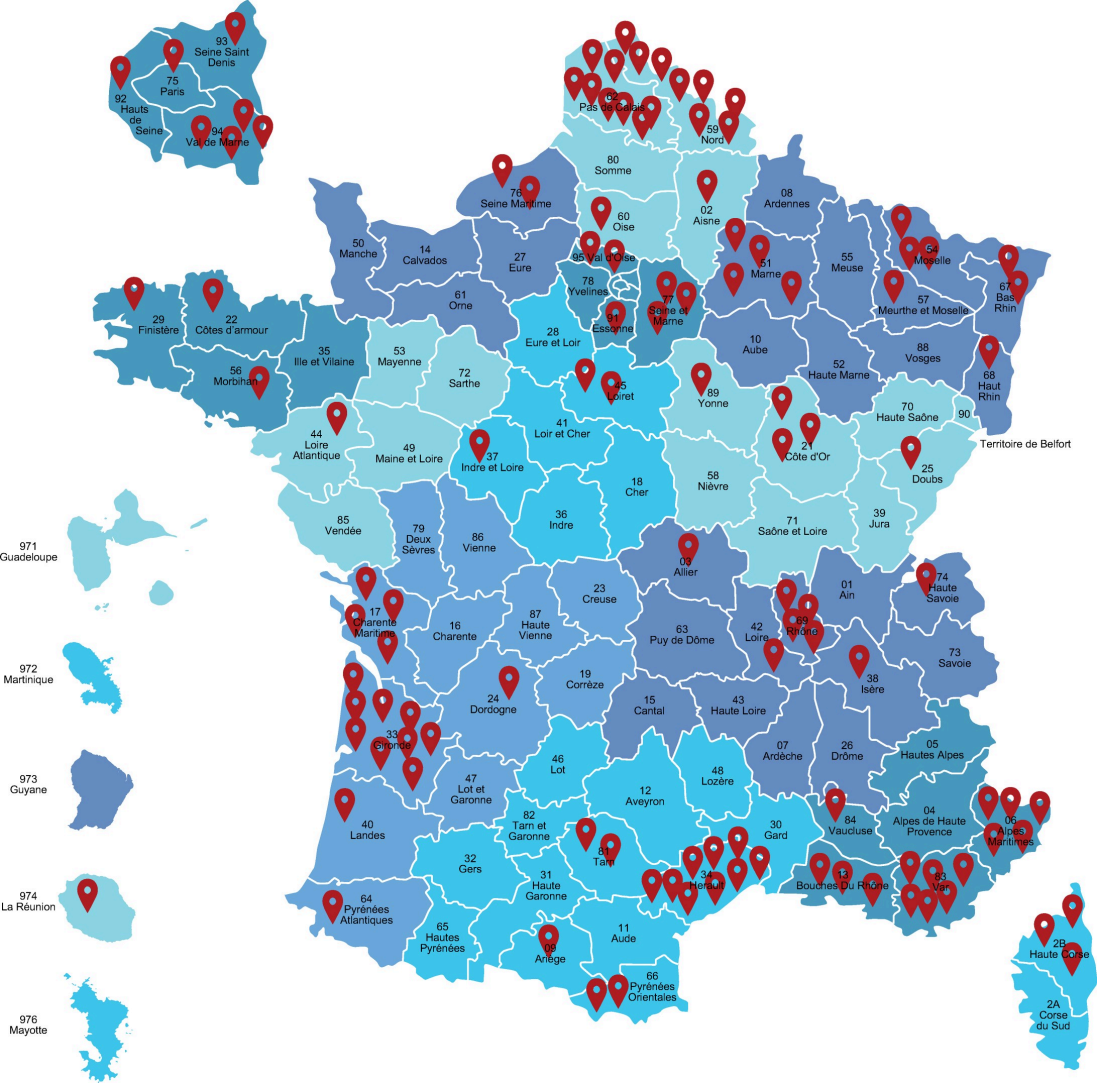
Regional distribution of survey respondents across France.

The survey was distributed via email to 563 private practice pulmonologists, of which 116 (20.6%) responded and 88 (15.6%) provided complete responses. All questions and responses are presented in **S1 File**. Most respondents (47.4%) were between 55 and 65 years of age (**Fig 1 and S1 Fig in S1 File**) and were not only based in private practice, but also saw patients in private clinics (49.1%), University Hospitals (13.2%) or non-University Hospitals (20.2%); only 17.5% were based solely in private practice. Regionally, most respondents were located in the Northern, South-Western and South-Eastern regions of France, with an over-representation in the Hauts-de-France region (**Fig 2 and S1 Fig in S1 File**).

### COPD management in clinical practice

Of the respondents, 33.0% reported seeing over 50 patients with COPD per month, 49.1% reported seeing 25–50 patients per month, 17.0% reported seeing 10–25 patients per month and just 0.9% reported seeing <10 patients per month. Overall, 87.4% of respondents stated that the management of COPD represented over 15% of their total clinical activity (**S2 Fig in S1 File**).

### Patient care pathway for COPD exacerbations

With regard to COPD exacerbations, 65.1% of respondents reported seeing ≥5 cases per month (**S2 Fig in S1 File**). Most respondents also received 2–5 or 5–10 calls per month from both general practitioners (GPs) and patients relating to exacerbations (55.4% and 59.8%, respectively; **S3 Fig in S1 File**).

Almost all patients who experienced an exacerbation were initially treated as outpatients (97.0%). Indeed, for most respondents (76.8%), outpatients accounted for over half of their treated patients (**S4 Fig in S1 File**).

For patients who were hospitalized due to a COPD exacerbation, 52.2% were initially treated in a private hospital and 49.4% in a public hospital, with 62.8% being treated in an emergency department (in a private or public hospital; **S4 Fig in S1 File**). The most common reasons for hospitalization due to an exacerbation were associated comorbidities (67.4%), oxygen saturation <85% (65.3%) and neurological disorders such as headaches and drowsiness (65.3%) (**S3 Fig in S1 File**).

In terms of initial outpatient assessments, the most frequently performed etiological assessments were: patient history (100%), medication survey (adherence, misuse, discontinuation; 98.9%), exacerbation history including severity (97.9%) and infection screening (96.8%). The most frequently performed blood panels were: full blood/platelet counts (86.6%), C-reactive protein (86.6%), B-type natriuretic peptide (68.9%) and blood electrolytes (Na, K, Cl; 64.1%). The most common respiratory function tests were: blood gas analysis (95.7%), plethysmography (62.5%), spirometry (61.2%) and reversibility testing (34.6%). The most frequently undertaken imaging modalities were: chest x-rays (88.2%), computerized tomography (CT) angiography (27.9%), chest CT scans (26.9%) and echocardiograms (21.1%). The most commonplace comorbidity screens performed were: cardiology consultations (58.2%), metabolic screening (29.0%) and ear, nose and throat consultations (26.6%). Across all areas, broadly similar trends were reported for hospital assessments, apart from bacteriological sampling which was performed more frequently in hospital compared with the outpatient setting (87.3% and 42.9%, respectively) (**S5 Fig in S1 File**).

First-line therapies typically used immediately post-exacerbation and in the outpatient setting are summarized in **Table 1**, and described in further details in **S6** and **S7 Figs in S1 File.** First-line therapies used in hospital were largely consistent with those used in the outpatient setting, but with a few notable differences. Long-acting muscarinic antagonist (LAMA), long-acting β_2_-agonist (LABA), inhaled corticosteroid (ICS)/LABA and LAMA/LABA therapies were used less frequently in hospital (36.4%, 33.3%, 20.5% and 36.4%, respectively) compared with the outpatient setting (59.2%, 57.7%, 38.2% and 58.2%, respectively) (**S7 Fig in S1 File**). Systemic corticosteroids, diuretics and low-molecular-weight heparin (LMWH) were used more often in hospital compared with the outpatient setting (hospital: 88.7%, 26.5%, 87.0%, respectively; outpatient: 68.5%, 14.5%, 5.8%, respectively) (**S7 Fig in S1 File**).

**Table 1:** Summary of first-line therapy for exacerbations in the outpatient setting and immediately post exacerbation.

	Initial prescription (outpatient), %^a^	Following an acute exacerbation, %^a^
**Inhaled therapies**		
**SABA**	91.7	85.7
**SAMA**	57.7	46.7
**LABA**	57.7	65.3
**LAMA**	59.2	42.4
**ICS/LABA**	38.2	51.9
**LABA/LAMA**	58.2	79.8
**Nebulization**		
**SABA**	90.9	64.0
**SAMA**	84.4	61.9
**Corticosteroids**	37.0	18.3
**Systemic corticotherapy**	68.5	21.7
**Antibiotic therapy**	87.9	40.5
**Diuretics**	14.5	5.9
**Bronchial drainage physiotherapy**	86.5	85.6
**Prevention LMWH**	5.8	9.3
**Mucomodulators**	19.8	11.4

^a^Percentage of respondents replying ‘yes’ to whether they prescribe a particular therapy in a particular setting (from a yes/no option).

ICS, inhaled corticosteroid; LABA, long-acting β_2_ agonists; LAMA, long-acting muscarinic antagonists; LMWH, low-molecular-weight heparin; SABA, short-acting β_2_ agonists; SAMA, short-acting muscarinic antagonists.

**Fig 3:**
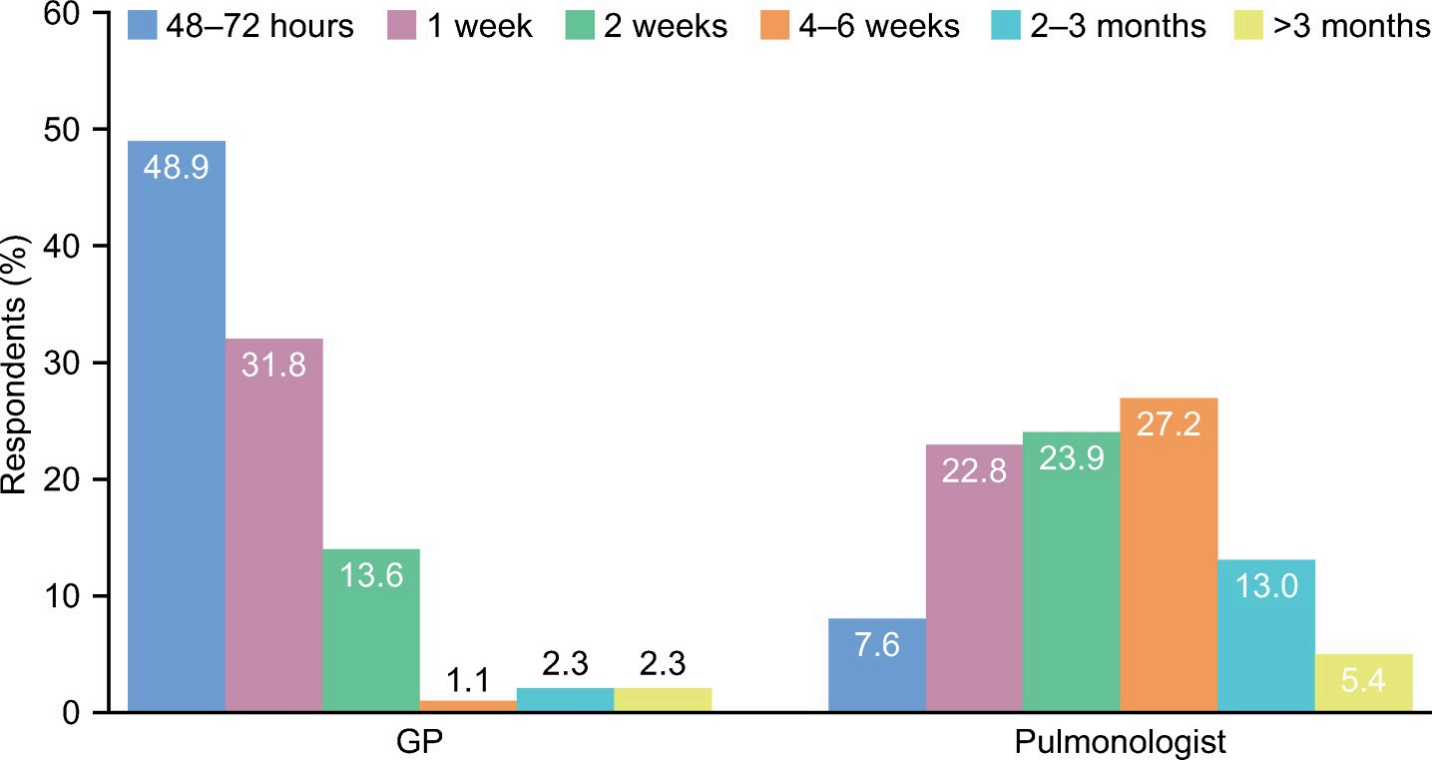
Time to follow-up following an acute COPD exacerbation. COPD, chronic obstructive pulmonary disease; GP, general practitioner.

Following an exacerbation, nearly all outpatient cases were followed-up within 2 weeks by the GP and within 1–6 weeks by the pulmonologist in line with HAS recommendations (94.3% and 81.5%, respectively; **Fig 3 and S8 Fig in S1 File**) [9]. Similar values were reported for hospital cases.

Overall, most respondents (76.7%) were in favor of establishing a support platform to coordinate the actions of all individuals involved in outpatient care (**S8 Fig in S1 File**).

### Secondary prevention of COPD exacerbations

**Fig 4:**
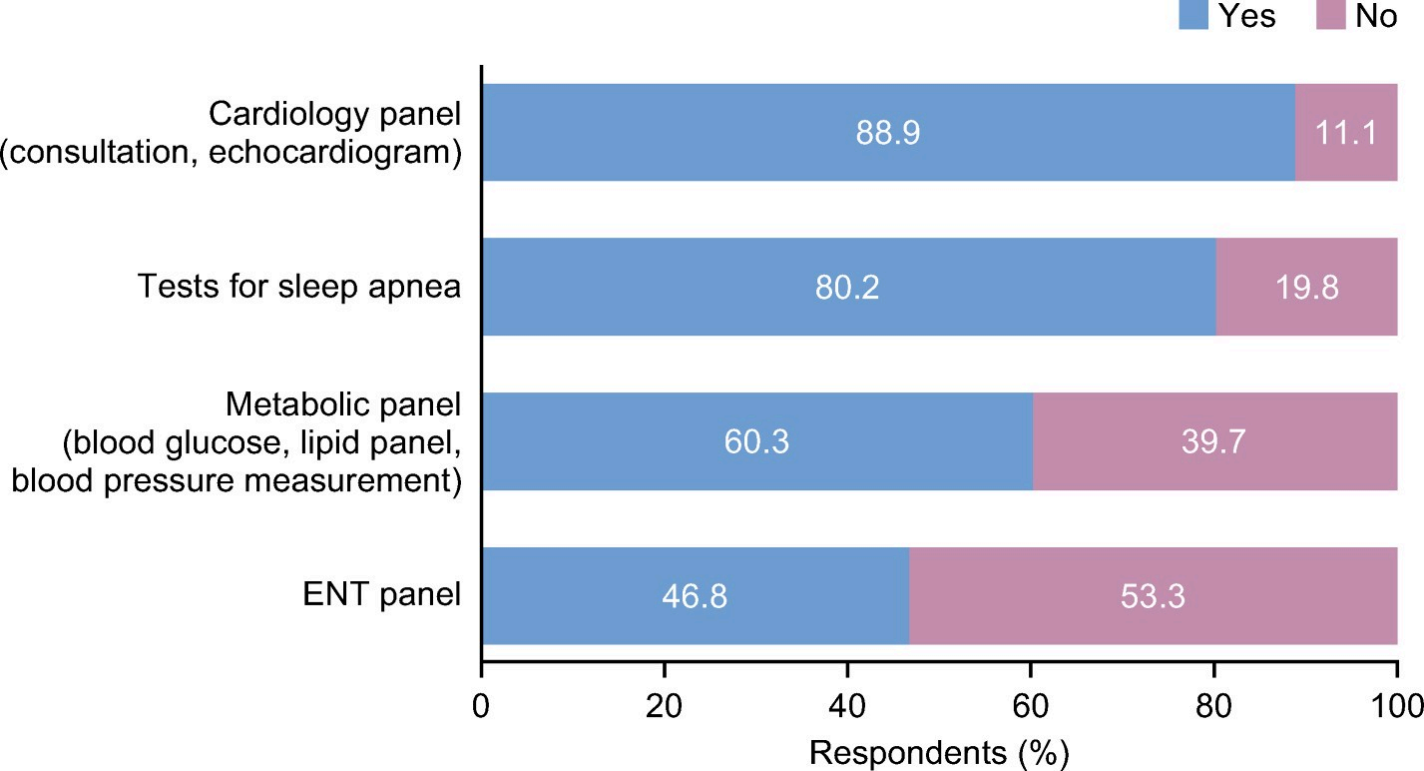
Comorbidity assessments implemented as secondary prevention measures in the outpatient setting^a^. ^a^Similar values were reported for hospital assessments. ENT; ear, nose and throat.

The most common secondary prevention measures implemented within the outpatient setting were: vaccination (98.9%), help with smoking cessation (95.4%), pulmonary rehabilitation (79.3%), comorbidity assessments (79.3%) and therapeutic education (65.1%) (**Fig 4 and S9 Fig in S1 File**). Similar values were reported within the hospital setting apart from the PRADO-BPCO home discharge care program: 52.4% of respondents said they would use this in the hospital setting compared with 14.7% in the outpatient setting (**S9 Fig in S1 File**).

**Fig 5:**
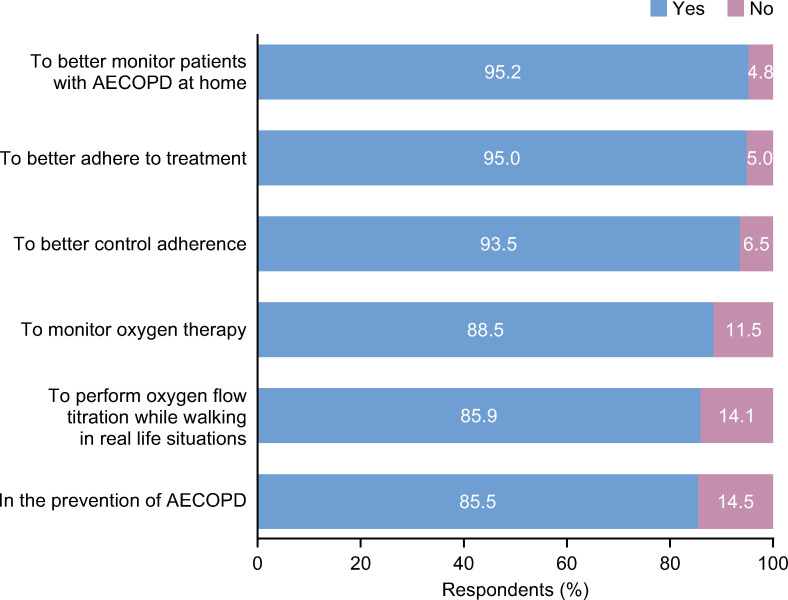
Key reasons respondents would use respiratory-connected devices. AECOPD, acute exacerbation of chronic obstructive pulmonary disease.

Most respondents (78.4%) were in favor of using respiratory-connected devices to follow-up patients at home. Key reasons for this preference included improving adherence to treatment, better monitoring of oxygen therapy and prevention of further exacerbations (**Fig 5 and S10 Fig in S1 File**).

## Discussion

The aim of this study was to investigate the management of COPD exacerbations within the private healthcare sector in France using a prospective, online, qualitative survey aimed at practicing pulmonologists. Overall, the results showed current clinical care practice to be in accordance with the recommendations of the SPLF and HAS [9, 10].

The management of COPD was shown to be a core clinical activity for practicing pulmonologists within the private healthcare sector. An ongoing, prospective, multicenter, observational study by Raherison *et al*. recently reported data relating to comorbidities and disease severity in patients with COPD from a clinic-based cohort in the South West of France (the Palomb cohort) [14]. The primary analysis of 1,584 patients indicated that the management of patients differed according to the comorbidities they experienced, despite being diagnosed with the same severity of COPD [14]. As part of the study, the investigators depicted five phenotypes according to the type of comorbidity: cardiac conditions, denutrition, osteoporosis, bronchiectasis and metabolic syndrome [14]. The results of our survey are consistent with the findings of Raherison *et al*. showing that multimorbidity is common within patients with COPD. Survey respondents also indicated that they work closely with GPs and a large multidisciplinary team to manage patients with COPD experiencing exacerbations, in line with current SPLF and HAS guidelines [9, 10].

Assessments performed in the outpatient and hospital settings were broadly similar, with some notable exceptions. The use of bacterial sampling was more frequent in the hospital setting compared with the outpatient setting, which could be reflective of differences in the availability of these types of expertise and services. The use of LAMA, LABA, ICS/LABA and LAMA/LABA as first-line therapies was more frequent in the outpatient setting, whereas systemic corticosteroids were used more frequently in hospitals. This is to be expected, as the current GOLD strategy involves the use of combinations of ICS, LAMA and LABA for the pharmacological management of COPD [1], and these agents would most likely be administered in the outpatient setting as part of routine care. Further, systemic corticosteroids should only be used as part of acute exacerbation management following hospitalization, in order to improve forced expiratory volume in 1 second oxygenation and shorten overall recovery time [1]. LMWH was rarely used in the outpatient setting compared with the hospital setting. This was anticipated as patients requiring LMWH are likely to be bedridden (i.e. hospitalized).

The implementation of the PRADO-BPCO home care program was less frequent in the outpatient setting compared with the hospital care setting. The program is a home discharge care package which aims to reduce readmission rates of patients with COPD following an acute exacerbation [12]. Following hospital discharge the program involves a coordinator performing a home visit followed by the arrangement of outpatient appointments to follow-up patients effectively and within a set timeframe [12]. The clear difference in the frequency of PRADO-BPCO program use between outpatient and hospital settings may reflect differences in knowledge and/or availability, and further education outside the hospital setting may be warranted.

Most participating pulmonologists were in favor of using respiratory-connected devices to improve home care, treatment adherence and secondary prevention of exacerbations. This information could serve to encourage the future development of implantable and wearable devices for COPD management and promote their integration into future disease management strategies.

There are both strengths and limitations of this study that should be considered when interpreting the results. The findings from this study provide new information on COPD management within a real-world setting, as well as important insights into the clinical practice preferences of private pulmonologists outside of public hospitals in France. Limitations include the age range, as over half of the survey respondents were >55 years of age. This is in contrast to the Direction de la Recherche, des Études, de l’Évaluation et des Statistiques (DREES) 2018 data [15], which reported that approximately 31% of pulmonologists in France were between 55 and 65 years of age, indicating a potential age bias towards the more senior pulmonologists in this study. As the survey captured data concerning the real-life practices of physicians, there was the potential for recall bias within the study. Responses were received from only 113 pulmonologists and this small sample size may limit the applicability of these findings to the private practice setting. Furthermore, as this research covers only the perspective of the pulmonologist, it would be advantageous in the future to also capture patient views on their experience of their COPD management in the private practice setting in France. Other limitations include those inherent to online survey studies, such as responder bias, question selection/incomplete information (some questionnaires were not fully completed) and sampling issues, potentially reflected in the geographical clusters for respondents in the Northern, South Western and South Eastern regions of France.

## Conclusion

The management of COPD and COPD exacerbations forms part of the core activity of private practice pulmonologists in France, who work closely with multidisciplinary teams as part of healthcare plans in line with SPLF and HAS guidelines. The use of respiratory-connected devices was highlighted as being an important new strategy for improving patient care following an exacerbation, particularly for patients with multiple comorbidities.

## Supporting information

S1 File (DOCX)

S1 Data (XLSX)
