# Supplementary material for: Management of acute COPD exacerbations in France: A qualitative survey in a private practice setting
Source: PLoS One. 2021 Jan 22;16(1):e0245373. doi: 10.1371/journal.pone.0245373 (PMC7822540; doi:10.1371/journal.pone.0245373)

**S1 Fig. Respondent profile.**


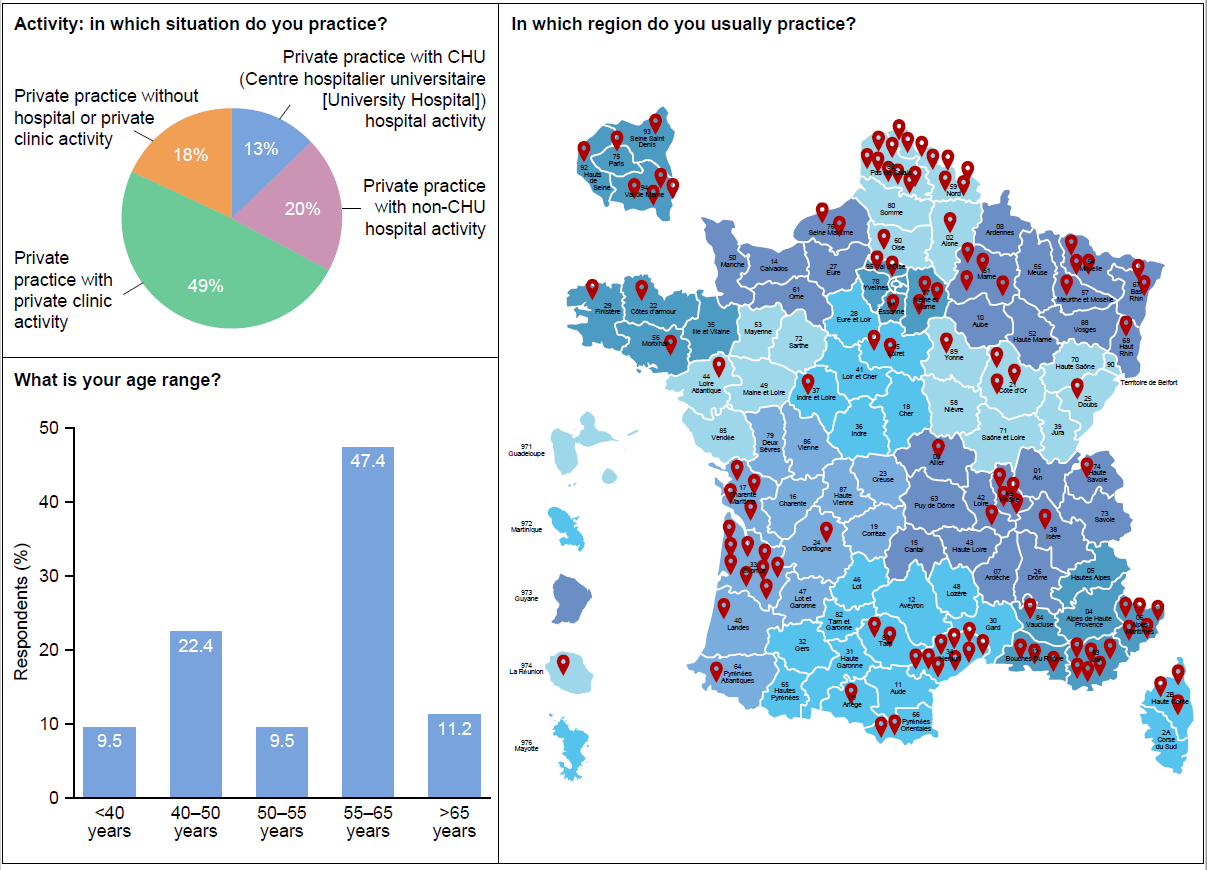


**S2 Fig. Place of COPD management in the activity.**


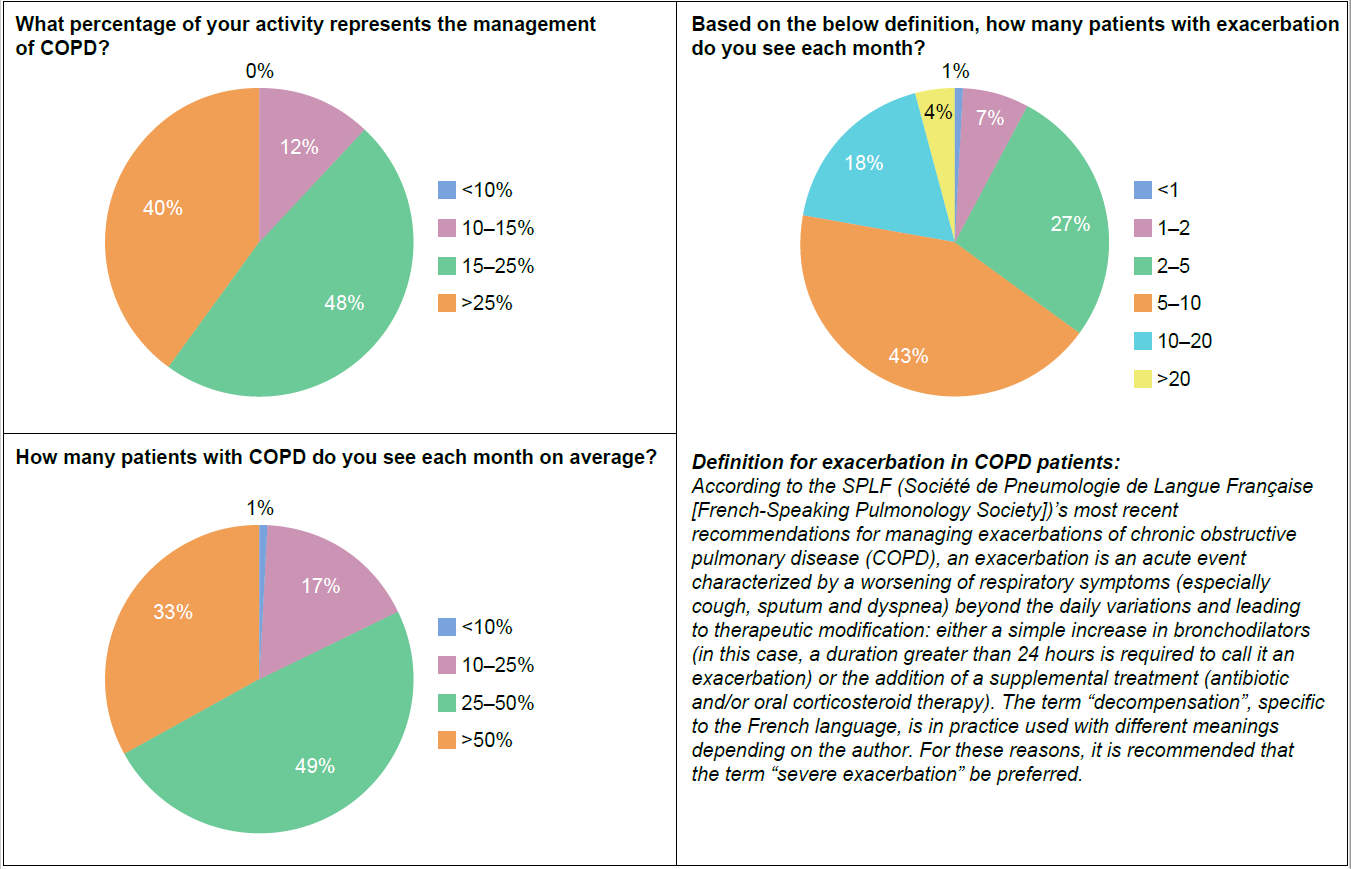


**S3 Fig. Patient care pathway in COPD exacerbation – subsequent activity.**


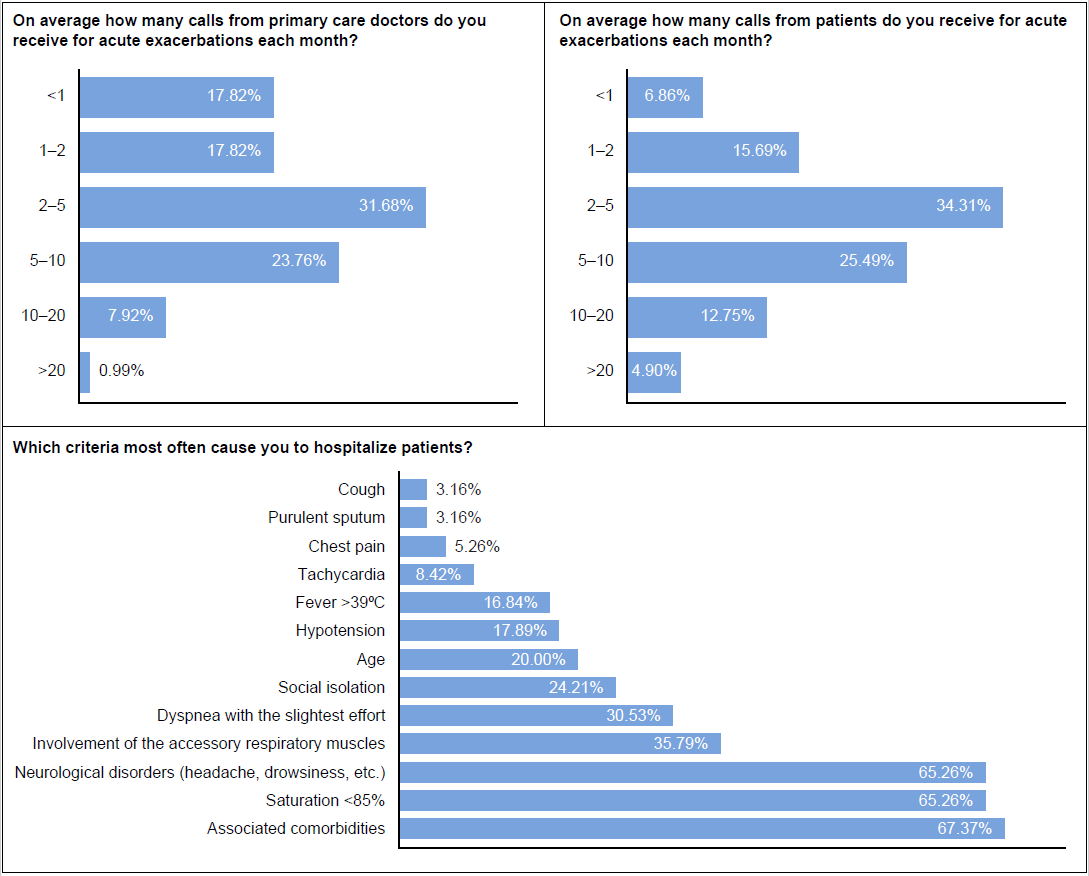


**S4 Fig. Patient care pathway in COPD exacerbation – place of initial treatment.**


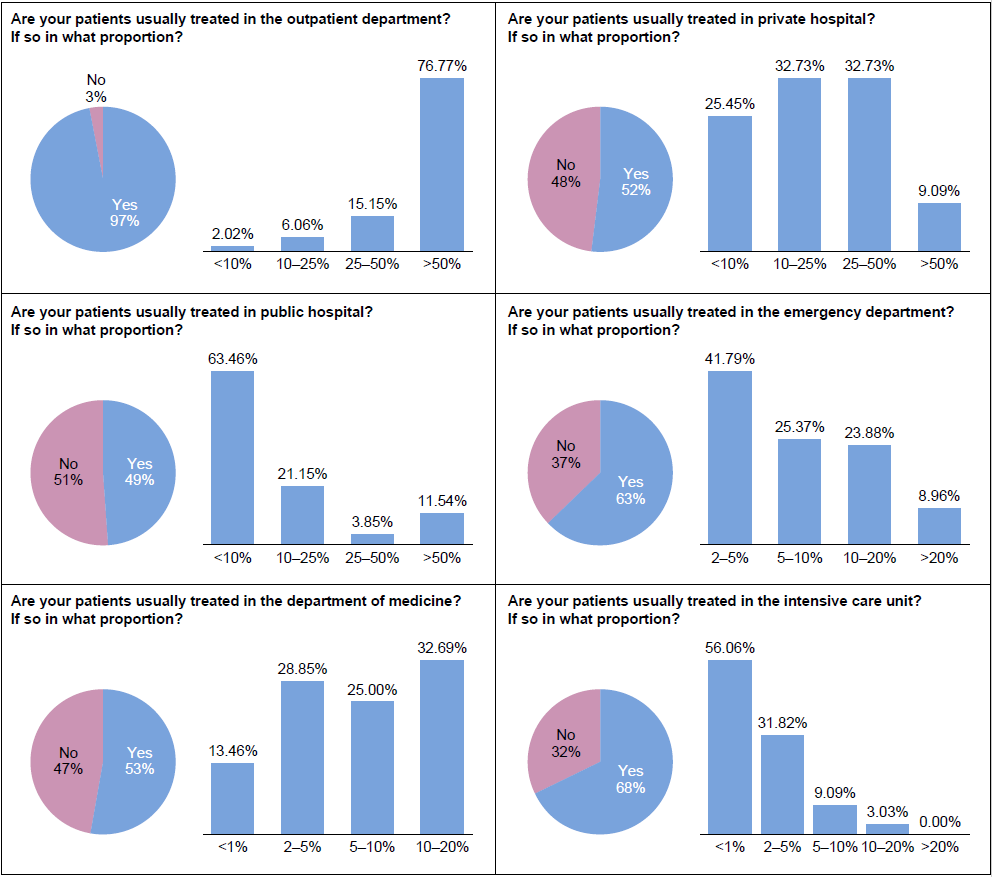


**S5 Fig. Patient care pathway in COPD exacerbation – assessments at time of exacerbation.**

**
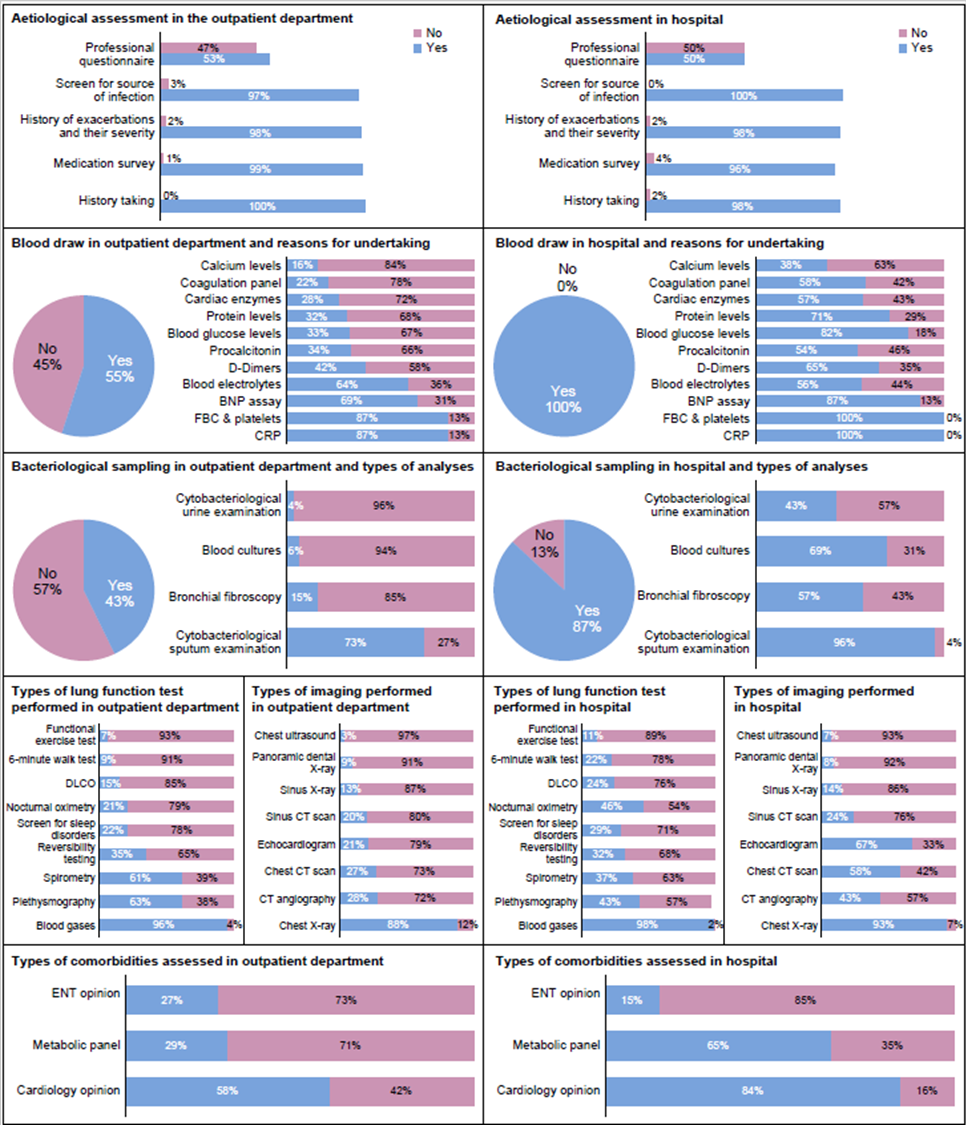
**

**S6 Fig. Patient care pathway in COPD exacerbation – standard treatment after acute exacerbation.**


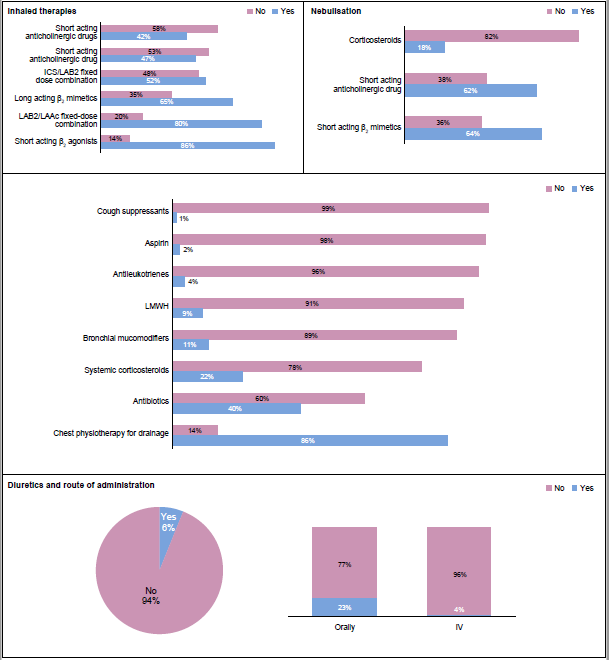


**S7 Fig. Patient care pathway in COPD exacerbation – standard first-line prescription.**


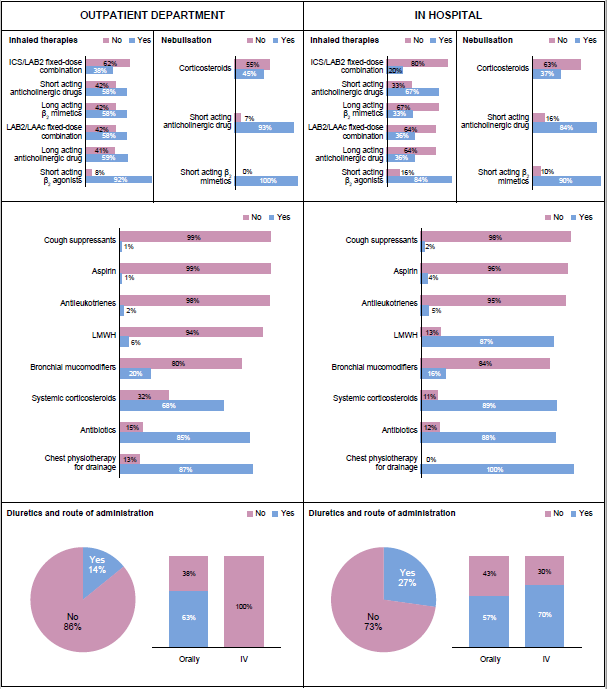


**S8 Fig. Secondary prevention measures – follow-up by healthcare professionals subsequent to acute exacerbation.**


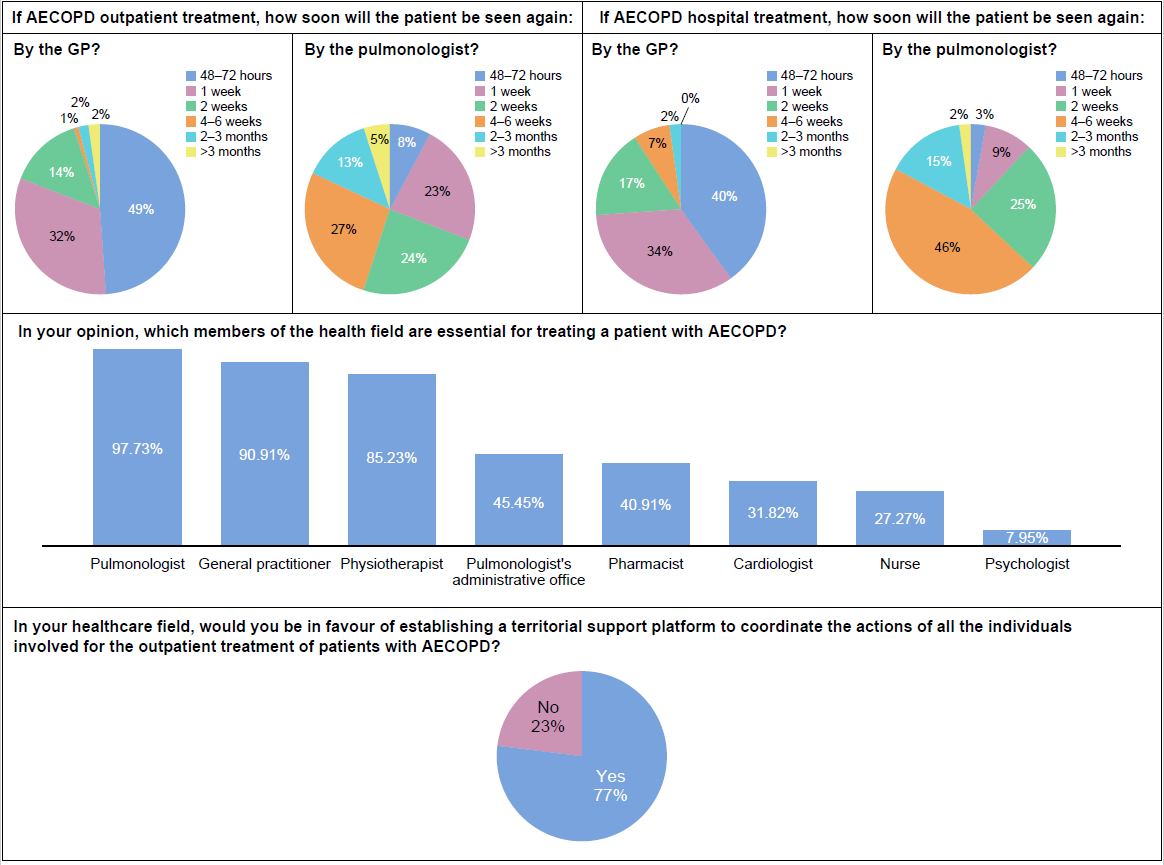


**S9 Fig. Secondary prevention measures established subsequent to acute exacerbation.**


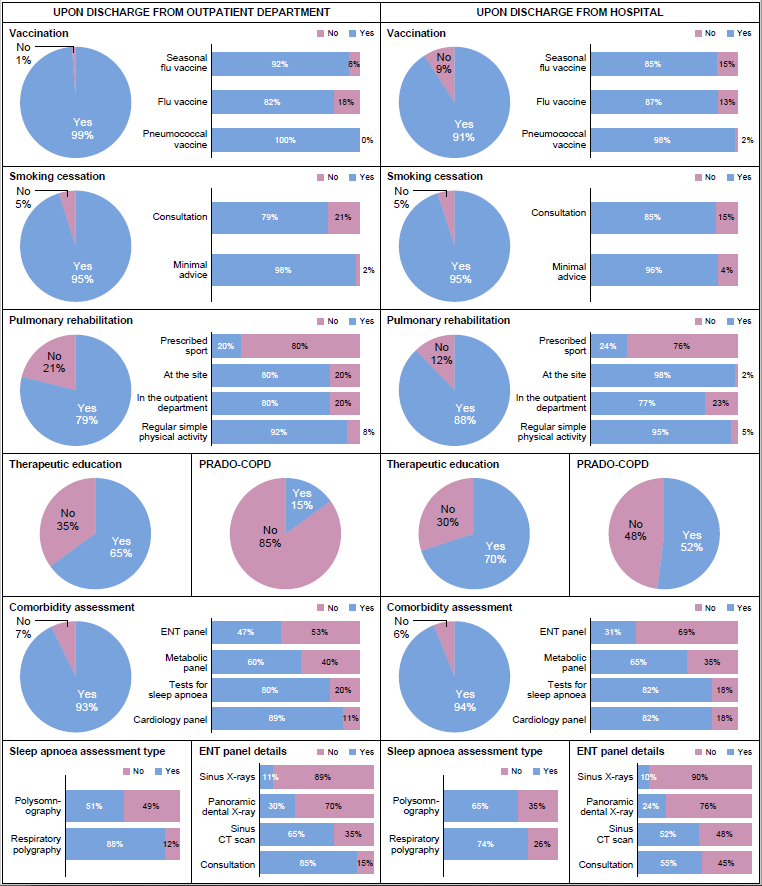


**S10 Fig. Secondary prevention measures – use of respiratory connected devices.**


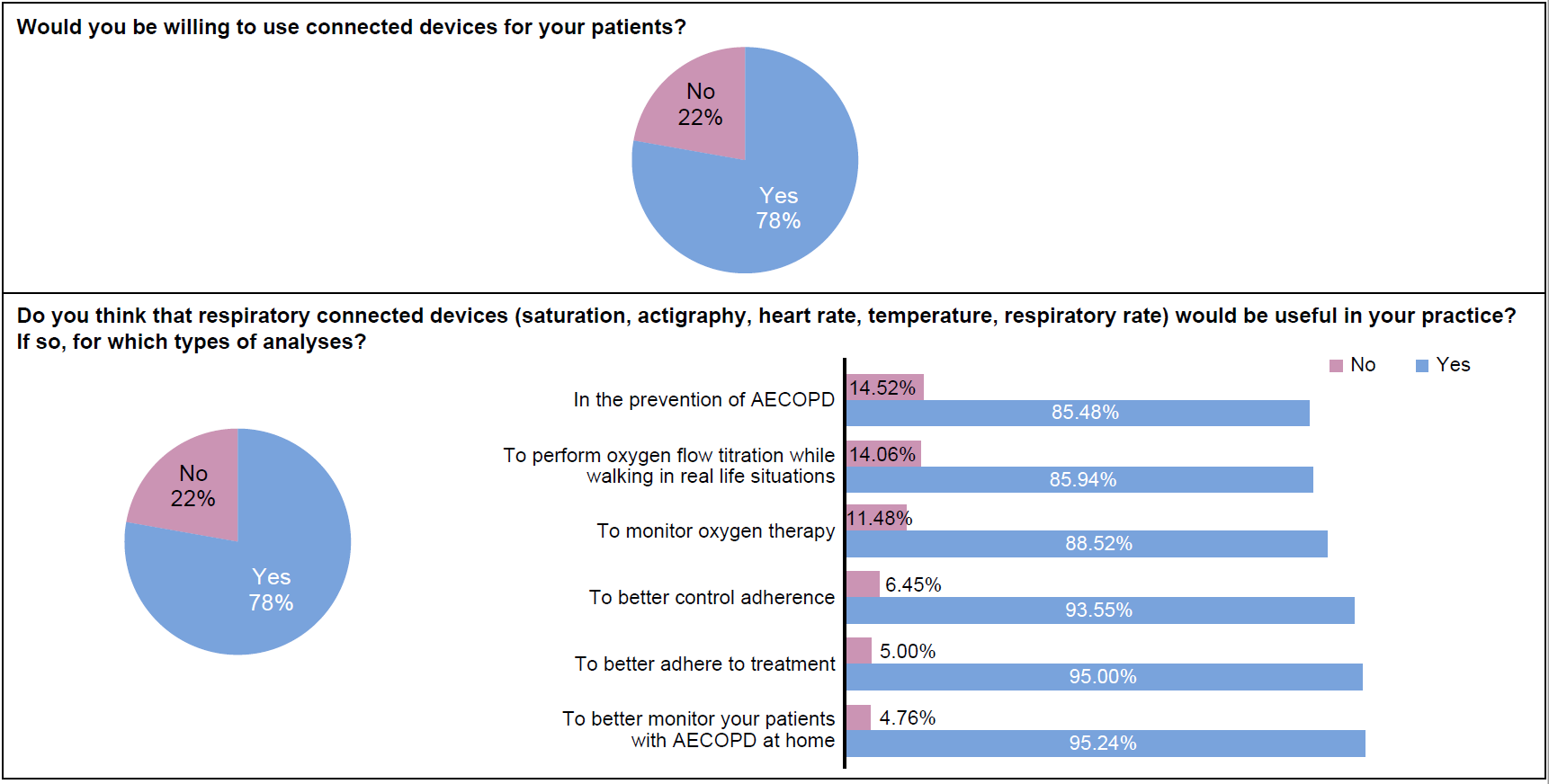

Supplement: S1 File — (DOCX) [file pone.0245373.s001.docx]
